# Dataset on the assessment of water quality of ground water in Kalingarayan Canal, Erode district, Tamil Nadu, India

**DOI:** 10.1016/j.dib.2020.106112

**Published:** 2020-08-01

**Authors:** R Divahar, P S Aravind Raj, S P Sangeetha, T Mohanakavitha, T Meenambal

**Affiliations:** a Aarupadai Veedu Institute of Technology, VMRF, Paiyanoor, Chennai 603104, India; b Tamilnadu Police Housing Corporation Limited, India; c School of Civil Engineering and Architecture, Adama Science and Technology University,Ethiopia

**Keywords:** Water quality index, Ground water, Kalingarayan canal, Physico chemical parameters

## Abstract

This data article aimed to investigate the quality of ground water in Kalingarayan Canal for the analysis of pollution level, Tamil Nadu. In order to understand the pollution status of the canal, nine ground water samples (GW1- GW9) were collected from the downstream side of the canal during the period between January 2014 – December 2016. Nine stations were selected along the Kalingarayan Canal, and ground water samples were collected on a monthly basis from these stations. The parameters like pH, electrical conductivity (EC), total dissolved solids (TDS), chlorides, total hardness (TH) nitrates, sulphates, sodium, calcium and magnesium were analyzed to observe the current status of the groundwater quality. Also, the groundwater quality is expressed in terms of Water Quality index (WQI). The APHA method was applied to determine the physico chemical parameters of the water samples. From the investigation, WQI reflects a low quality of groundwater in sampling stations Kolathupalayam (GW3) and Perumparai (GW6) which is mainly contaminated with nitrate and the water is found to be very hard in nature. Also, it was observed that calcium and magnesium content in groundwater is very high at certain stations. Most of the groundwater from this place cannot be used for any kind of industrial processes and human consumption without proper treatment.

## Specifications table

**Table:** 

**Subject**	Environmental Engineering
**Specific subject area**	Water Quality
**Type of data**	Table Figure
**How data were acquired**	All experiments were done using titrimetric testing for temporary and permanent hardness, calcium, magnesium and chloride. System testing also included pH (WTW model) and electrical conductivity (ESI model). The analysis of sulfate anions and cations was done by spectrophotometry (DR5000; Hach) in water. The total hardness and TDS were determined by the EDTA titrimetric method and gravimetry, respectively.
**Data format**	Raw Analyzed
**Parameters for data collection**	All water samples were collected in polyethylene bottles and stored in an ice-jacket placed at a 4°C room temperature
**Description of data collection**	Water Quality Index and Physico chemical parameters of Kalingarayan Canal
**Data source location**	City/Town/Region: Kalingarayan Canal, Erode District, Tamilnadu Country: India
**Data accessibility**	With the article
**Related research article**	T. Mohanakavitha and T. Meenambal, Assessment of water quality index for the groundwater in downstream side of the Kalingarayan canal, erode district, Tamilnadu state, India, Pollution Research, 32(2), 2013, pp. 245-249. [1]

## Value of the data

 

• The data provided in this article reflect the analysis of pollution level of the Kalingarayan Canal.
• Determination of the levels of the physical and chemical parameters of pH, electrical conductivity (EC), total dissolved solids (TDS), chlorides, total hardness (TH), nitrates, sulphates, sodium, calcium and magnesium were analyzed to observe the current status of the ground water quality of the Kalingarayan Canal country in India.
• Water quality index (WQI) is one of the most effective tools to communicate information about the quality of water to the citizens concerned and policy makers. Hence it becomes important to assess and manage the ground water quality.
• This data will be useful to the society, since groundwater is one of the most important source of drinking water. It is also useful to reach the socio-economic objectives like income, production and quality of life. This information provided can be extended to other canals for analysis of groundwater quality.
• The data can potentially make an impact on society. As there is a rapid growth in industrialization, the water body along the river gets polluted. This data provides the level of pollution and its environmental impact interms of short and long term. Also, It can be useful in the context of regional planning.
• The result of analysis of the data shows that the water in this area is not desirable for industrial processes and human consumption without proper treatment.
• The ground water is contaminated mainly with nitrate. The water is very hard in nature at certain locations due to high concentration of calcium and magnesium content in groundwater during the three years, indicating that most of the ground water locations were not suitable for irrigation purposes.

## 1 Data description

**Fig. 1:**
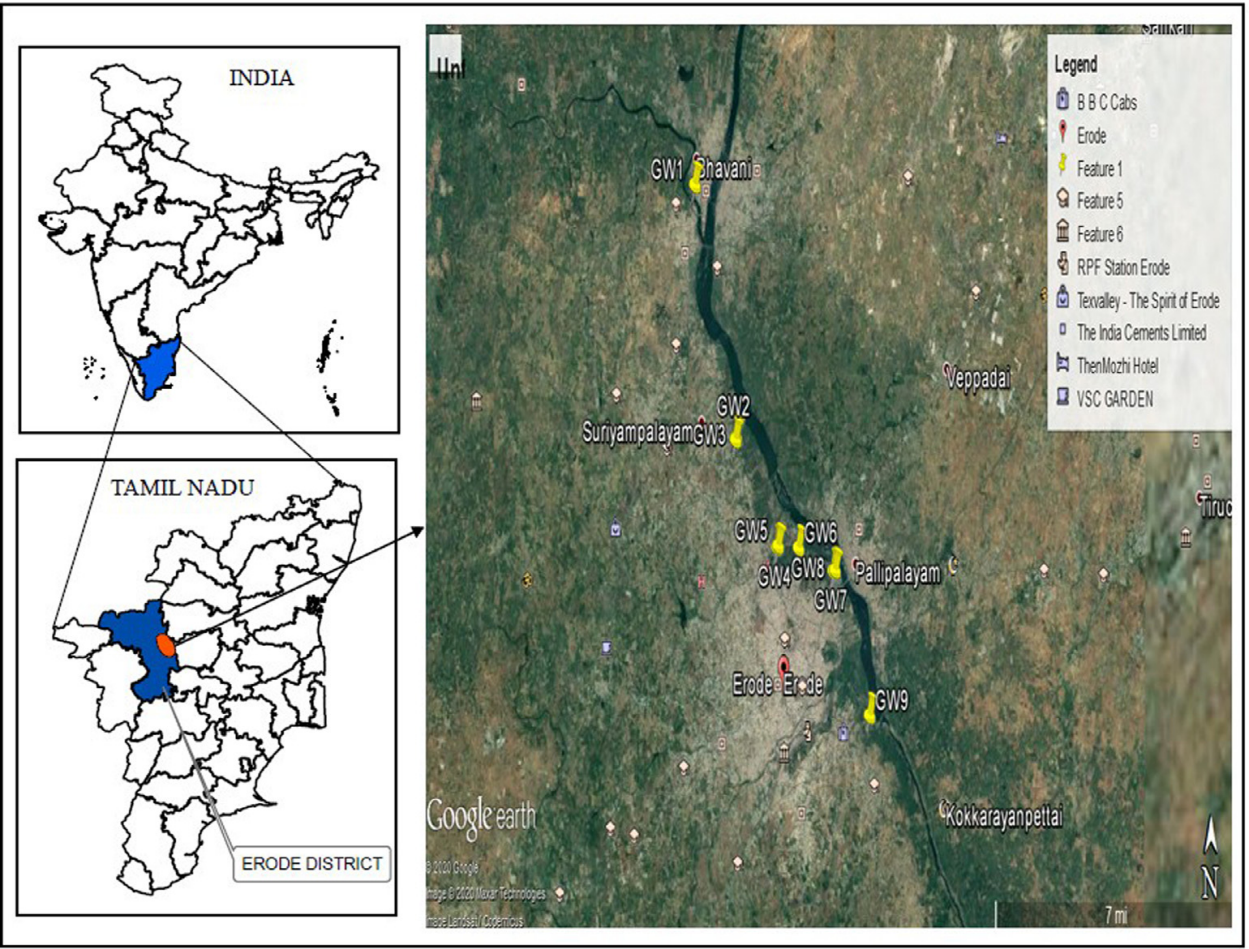
Locations of the monitoring stations in Kalingarayan Canal (Modified from Divahar et al. [3]).

The construction work of Kalingarayan Canal was carried out during the period 1271 AD–1283 AD. The canal starts with a Kalingarayan dam on River Bhavani, near Bhavani and flows through Erode before terminating near Kodumudi. It is designed in a circuitous way with as many twists and turns as possible. The canal is in the curvilinear path to cover more land area for irrigation. The length of the canal is 92 km passing entirely through the Erode district, Tamil Nadu as per the survey conducted. The mean sea level (MSL) where the canal begins is 534 feet and ends at 412 feet. The Kalingarayan Canal is situated on the western bank of the river Cauvery at 77° 40´ E to 77° 48´ E longitude and 11° 16´ N to 11° 26´ N with an area of 7621 Sq. km. Based on the latest population census in Erode district (2011), its population was 521,900. There are number of tannery and textile industries located across the river which spoils the quality of river by discharging its effluent into the river water, which inturn spoils the ground water quality in the surrounding areas. The data contain analysis of pollution level of group water samples [[2], [3], [4]–5]. Nine ground water samples (GW1- GW9) were collected from the downstream side of the canal during the period between January 2014 – December 2016 and ground water samples were collected on a monthly basis from these stations. The parameters like pH, electrical conductivity (EC), total dissolved solids (TDS), chlorides (Cl), total hardness (TH), nitrates (NO_3_^−1^), sulphates (SO_4_^−2^), sodium(Na), calcium(Ca) and magnesium(mg) were analyzed to observe the current status of the groundwater quality. Also, the groundwater quality is expressed in terms of Water Quality index (WQI). The APHA method was applied to determine the physico chemical parameters of the water samples. The data set pertaining to pH, EC, and physico chemical parameters, are shown in Table 2-10. The details of groundwater sampling in nine different locations in the Kalingarayan Canal (Table 1, Fig. 1).

**Table 1.** Location of ground water samples in Kalingarayan Canal.

S. No.	Latitude	Longitude	Sample Code	Distance From the Canal (m)	Sampling Location
1	11°26′26.37"N	77°40′41.27"E	GW1	90	Panjalingapuram towards north direction
2	11°23′12.49"N	77°41′43.73"E	GW2	170	Kolathupalayam I towards south direction
3	11°23′14.52"N	77°41′45.09"E	GW3	30	Kolathupalayam II towards north direction
4	11°21′50.78"N	77°42′46.87"E	GW4	100	Unjalur I towards north-east direction
5	11°21′51.86"N	77°42′49.24"E	GW5	150	UnjalurII (hospital) towards north-east direction
6	11°21′50.21"N	77°43′20.56"E	GW6	50	Perumparai towards north-east direction
7	11°21′32.69"N	77°44′13.54"E	GW7	85	Vadakupudupalayam I towards north direction
8	11°21′33.29"N	77°44′16.51"E	GW8	200	Vadakupudupalayam II towards north direction
9	11°19′42.82"N	77°45′9.44"E	GW9	150	Sallikadu towards north direction

## 2 Experimental design, materials and methods

### 2.1 Materials and methods

Ground water samples were collected from open wells at nine different locations in the surrounding irrigation fields over a period of three years from 2014 to 2016 once in a month. The groundwater samples were collected throughout the year (at the time of flow, i.e during monsoon season, since the river is mostly fed by the southwest monsoon [4] and at the time of non-flow of water, i.e during the summer season in the canal). The bottles were washed with detergent and dilute nitric acid before sampling. Finally, the de-ionized water was used to rinse the sampling bottles and the dried in sunlight. Samples were collected in plastic bottles from each sampling point. The groundwater samples were coded as GW. The details of groundwater sampling in nine different locations in the Kalingarayan Canal (Table 1, Fig. 1). The reason behind this is that the effluents are discharged into the canal during the flow period. But during non-flow period, it is not possible to discharge the effluents into the canal and it is discharged into the ground. This pollutes the groundwater. The basic water quality parameters were analyzed using the analytical methods are shown in Table 13 [[6], [7], [8]–9]. The parametric values are compared year wise for individual sampling station (flow and non-flow period) and the values are shown in Tables 2 to 10 respectively. In this sampling station all the parameters are within the permissible limit of that of the drinking water range except nitrate. In this region also, water is contaminated with nitrate during the non-flow period of the canal. This indicates that the reduction in groundwater table makes water, insufficient for dilution of these contaminants. By using an ion exchange denitrification process, excess nitrates can be removed easily.

**Table 2.** Physico-chemical parameters of groundwater sample at GW1 a distance of 90m away from the canal.

Parameters	At the time of flow(Year)				At the time of non-flow(Year)			
	2014	2015	2016	SD	2014	2015	2016	SD
pH	6.40	6.80	6.66	0.20	7.40	7.90	7.80	0.26
EC(µS/cm)	906	956	1094	97.37	1001	1076	1263	134.93
TDS(mg/L)	580	612	700	62.13	640	688	808	86.53
Chloride (mg/L)	269	289	338	35.28	306	324	376	36.24
Sulphate (mg/L)	59	63	85	13.94	76	80	102	14.00
Sodium (mg/L)	95	102	113	8.86	108	114	125	8.51
Calcium (mg/L)	97	104	101	3.54	106	112	110	3.09
Magnesium(mg/L)	33	35	51	9.81	30	35	57	14.53
Hardness (mg/L)	377	405	462	43.54	389	424	511	62.59
Nitrate (mg/L)	22.8	27.4	25.7	2.33	25.4	26.9	28.2	1.40

**Table 3.** Physico-chemical parameters of groundwater sample at GW2 a distance of 170m away from the canal.

Parameters	At the time of flow(Year)				At the time of non-flow(Year)			
	2014	2015	2016	SD	2014	2015	2016	SD
pH	6.40	6.80	6.70	0.21	7.60	8.10	7.93	0.25
EC(µS/cm)	1071	1138	1263	97.45	1206	1347	1469	131.61
TDS(mg/L)	686	728	809	62.52	772	862	940	84.07
Chloride (mg/L)	412	440	487	37.90	463	492	541	39.32
Sulphate (mg/L)	98	106	126	14.48	117	124	146	15.02
Sodium (mg/L)	27	30	41	7.60	36	38	51	8.03
Calcium (mg/L)	86	92	91	3.12	94	100	98	3.09
Magnesium (mg/L)	27	30	44	9.19	29	31	45	8.83
Hardness (mg/L)	327	354	407	40.92	356	378	431	38.39
Nitrate (mg/L)	28.2	32.2	31.1	2.07	34.3	36.4	37.2	1.50

**Table 4.** Physico-chemical parameters of groundwater sample at GW3 a distance of 30m away from the canal.

Parameters	At the time of flow(Year)				At the time of non-flow(Year)			
	2014	2015	2016	SD	2014	2015	2016	SD
pH	7.20	7.60	7.56	0.22	8.20	8.70	8.53	0.25
EC(µS/cm)	3581	3799	3759	116.05	3947	4198	4200	145.50
TDS(mg/L)	2292	2431	2406	74.10	2526	2687	2688	93.24
Chloride (mg/L)	801	853	841	27.14	882	938	926	29.54
Sulphate (mg/L)	679	721	711	21.94	744	791	779	24.39
Sodium (mg/L)	571	607	598	18.80	630	670	661	20.90
Calcium (mg/L)	174	185	182	5.62	191	203	199	6.11
Magnesium (mg/L)	63	68	94	16.47	68	73	101	17.73
Hardness (mg/L)	695	742	838	72.89	757	805	911	78.96
Nitrate (mg/L)	32.8	37.0	36.1	2.21	38.2	40.7	41.1	1.57

**Table 5.** Physico-chemical parameters of groundwater sample at GW4 a distance of 100m away from the canal.

Parameters	At the time of flow (Year)				At the time of non-flow (Year)			
	2014	2015	2016	SD	2014	2015	2016	SD
pH	6.50	6.80	7.27	0.39	7.50	7.90	8.30	0.40
EC(µS/cm)	1084	1156	1847	421.27	1317	1362	1875	309.99
TDS(mg/L)	694	740	1182	269.45	843	872	1200	198.27
Chloride (mg/L)	186	214	415	124.80	228	243	365	75.14
Sulphate (mg/L)	136	145	285	83.72	171	182	267	52.65
Sodium (mg/L)	178	190	275	53.00	212	226	275	33.02
Calcium (mg/L)	132	140	149	8.35	146	155	169	11.32
Magnesium (mg/L)	38	40	62	13.20	42	44	69	15.16
Hardness (mg/L)	484	516	625	74.03	536	570	705	89.33
Nitrate (mg/L)	29.5	32.6	32.4	1.73	37.2	39.6	40.1	1.55

**Table 6.** Physico-chemical parameters of groundwater sample at GW5- a distance of 150m away from the canal.

Parameters	At the time of flow (Year)				At the time of non-flow (Year)			
	2014	2015	2016	SD	2014	2015	2016	SD
pH	6.90	7.30	7.40	0.26	7.60	8.10	8.29	0.36
EC(µS/cm)	1207	1337	1976	411.62	1480	1573	1795	161.84
TDS(mg/L)	772	856	1265	263.75	947	1007	1149	103.74
Chloride (mg/L)	233	288	462	119.43	297	316	316	10.88
Sulphate (mg/L)	136	145	281	81.47	176	187	180	5.62
Sodium (mg/L)	205	219	310	56.73	240	255	295	28.43
Calcium (mg/L)	132	140	150	8.86	145	155	192	24.48
Magnesium (mg/L)	36	38	59	12.45	40	43	71	17.33
Hardness (mg/L)	477	508	614	71.95	527	561	771	132.39
Nitrate (mg/L)	29.2	32.0	32.1	1.65	44.4	46.8	46.9	1.42

**Table 7.** Physico-chemical parameters of groundwater sample at GW6 a distance of 50m away from the canal.

Parameters	At the time of flow (Year)				At the time of non-flow (Year)			
	2014	2015	2016		2014	2015	2016	
pH	7.40	7.80	7.71	0.21	8.10	8.60	8.45	0.26
EC(µS/cm)	3256	3464	3383	104.84	3586	3814	3821	133.70
TDS(mg/L)	2084	2217	2165	67.02	2295	2441	2446	85.77
Chloride (mg/L)	770	820	808	26.02	842	895	884	27.97
Sulphate (mg/L)	742	786	776	23.07	812	863	850	26.50
Sodium (mg/L)	296	315	316	11.15	339	361	361	12.70
Calcium (mg/L)	199	212	208	6.60	213	227	223	7.18
Magnesium (mg/L)	66	71	98	17.27	73	77	107	18.76
Hardness (mg/L)	770	822	921	76.60	831	884	997	84.73
Nitrate (mg/L)	31.4	36.1	34.3	2.37	37.0	39.4	39.9	1.55

**Table 8.** Physico-chemical parameters of groundwater sample at GW7- a distance of 85m away from the canal.

Parameters	At the time of flow(Year)				At the time of non-flow(Year)			
	2014	2015	2016	SD	2014	2015	2016	SD
pH	7.00	7.50	7.57	0.31	7.50	7.90	8.28	0.39
EC(µS/cm)	1092	1166	1505	220.21	1379	1467	1571	96.11
TDS(mg/L)	699	747	963	140.63	883	939	1005	61.07
Chloride (mg/L)	272	290	391	64.37	343	364	406	31.91
Sulphate (mg/L)	82	86	91	4.56	135	144	94	26.42
Sodium (mg/L)	131	140	189	31.21	166	176	196	15.22
Calcium (mg/L)	144	154	224	43.76	157	167	233	41.01
Magnesium (mg/L)	36	39	98	35.08	39	42	108	38.94
Hardness (mg/L)	508	544	963	253.06	554	590	1024	261.41
Nitrate (mg/L)	42.4	44.1	45.3	1.46	54.3	57.8	57.2	1.87

**Table 9.** Physico-chemical parameters of groundwater sample at GW8 a distance of 200m away from the canal.

Parameters	At the time of flow(Year)				At the time of non-flow(Year)			
	2014	2015	2016	SD	2014	2015	2016	SD
pH	6.50	6.90	7.04	0.28	6.90	7.40	7.65	0.38
EC(µS/cm)	918	1035	1354	225.66	1113	1197	1358	124.50
TDS(mg/L)	587	662	866	144.38	712	766	869	79.76
Chloride (mg/L)	239	277	402	85.45	269	284	396	69.34
Sulphate (mg/L)	91	98	115	12.23	136	148	113	17.79
Sodium (mg/L)	79	86	102	12.01	102	112	101	6.18
Calcium (mg/L)	124	132	168	23.55	135	145	166	15.55
Magnesium (mg/L)	31	33	62	17.58	33	36	61	15.60
Hardness (mg/L)	436	467	676	130.60	474	509	665	101.91
Nitrate (mg/L)	41.5	42.7	44.4	1.46	47.6	50.3	50.2	1.53

**Table 10.** Physico-chemical parameters of groundwater sample at GW9 a distance of 150m away from the canal.

Parameters	At the time of flow (Year)				At the time of non-flow (Year)			
	2014	2015	2016	SD	2014	2015	2016	SD
pH	6.50	6.80	6.91	0.21	7.00	7.50	7.50	0.29
EC(µS/cm)	965	1078	1435	245.33	1150	1192	1427	149.29
TDS(mg/L)	618	690	919	157.18	736	763	913	95.36
Chloride (mg/L)	265	306	431	86.53	293	311	444	82.42
Sulphate (mg/L)	94	101	173	43.73	125	133	125	4.53
Sodium (mg/L)	107	114	129	11.08	127	135	131	4.01
Calcium (mg/L)	98	104	118	9.99	105	112	131	13.18
Magnesium (mg/L)	26	28	48	12.17	28	30	53	14.07
Hardness (mg/L)	352	377	490	73.76	378	402	545	90.18
Nitrate (mg/L)	36.8	38.1	39.7	1.45	42.3	45.1	45.2	1.65

## 3 Analytical procedures

Water quality index (WQI) is a mathematical formula used in the assessment of overall quality of water by using the values of different water quality parameters. WQI is one of the most successful methods and it gives information on the quality of water. WQI was calculated using the World Health Organization standards [10] and Indian Standards [11] in the following steps. Water quality index method for groundwater quality assessment is widely used around the world for assessment & management of groundwater [[12], [13], [14]–15]. The WQI calculation was carried out using a weighted arithmetic index as shown below. The WQI calculations include three successive steps. Each of the 10 parameters has been assigned a weight (w_i_) according to its relative importance in the overall quality of water for drinking purposes. The maximum weight of 5 has been assigned to the parameter nitrate due to its major importance in water quality assessment. Magnesium which is given the minimum weight of 1 as magnesium by itself may not be harmful.

The first step is “assigning weight” each of the 10 parameters has been assigned a weight (w_i_) according to its relative importance in the overall quality of drinking water. The second step is the “relative weight calculation” calculated by following equation

$$W_{i}=\;\frac{w_{i}}{\sum_{i=1}^{n}w_{i}}$$

The third step is “quality rating (qi)” calculated by following equation

$$q_{i}=\frac{C_{i}}{S_{i}}\;x\;100$$

where, C_i_ is the concentration of each parameter in each water sample, S_i_ is the WHO standard value for each parameter. Finally, the W_i_ and q_i_ are used to calculate the SI_i_ for each parameters and then the WQI calculated from the following equation:

$$SI_{i}=W_{i}\;x\;q_{i}$$

$$WQI=\;\sum W_{i}q_{i}$$

where SI_i_ is the sub index of each parameter

Where both the summations are taken from i =1 to i= 10 (the total no. of parameters considered).

**Fig. 2:**
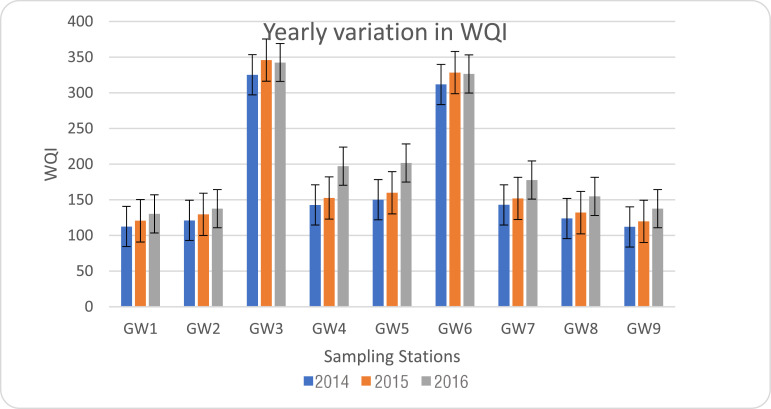
Variation in groundwater quality based on WQI.

WQI of ground water at each sampling point is shown in Table 12, Fig. 1. Table 11 deals with classification of drinking water quality. It was observed that 35% of groundwater samples are unsuitable for drinking and another 35% of samples are very poor water and remaining 30% of the samples are of poor category throughout the study period(2014, 2015 and 2016). The percentage of water samples under the ‘unsuitable for drinking’ category gradually increases. This indicates that the groundwater pollution in this region increases from 2014 to 2016. In the year 2015 and 2016, none of the samples fell under the good category which shows that all the groundwater samples have undergone some kind of pollution threats. In 2016, water samples that came under the category of very poor’ are 15.7%, while it is only 1.85% in the year 2015. So, the groundwater was contaminated more in the year 2016. Table 12. shows the quality of groundwater in each location with respect to Water Quality Index values during the study period. Fig. 2. shows the variation in the WQI values throughout the study period. During the study period, sampling stations GW3 and GW6 reach the maximum value while comparing with other sampling stations. So, the ground water withdrawn from these two wells could not be used for any purpose. In addition to that the sampling stations GW3 is located 30 m away from the canal and GW6 is located at 50 m away from the canal. The value of WQI increases with time in all the sampling stations. This gives a warning that the pollution of groundwater in that region must be prevented. The information obtained through this work may be used to improve the management practices and developing better water pollution control strategies for Kalingarayan Canal.

**Table 11.** Water quality classification based on WQI value.

WQI Value	Water Quality
<50	Excellent
50-100	Good Water
100-200	Poor Water
200-300	Very Poor Water
>300	Water Unsuitable For Drinking

**Table 12.** Quality of ground water based on WQI values.

Sample Code	2014		2015		2016	
	WQI values	Quality	WQI values	Quality	WQI values	Quality
GW1	113	Poor	121	Poor	130	Poor
GW2	121	Poor	130	Poor	138	Poor
GW3	325	Unsuitable for drinking	346	Unsuitable for drinking	343	Unsuitable for drinking
GW4	143	Poor	153	Poor	197	Poor
GW5	150	Poor	160	Poor	202	Very Poor
GW6	312	Unsuitable for drinking	329	Unsuitable for drinking	327	Unsuitable for drinking
GW7	143	Poor	152	Poor	318	Unsuitable for drinking
GW8	124	Poor	132	Poor	155	Poor
GW9	112	Poor	120	Poor	138	Poor

**Table 13.** Analytical methods of Water quality parameters.

S. No.	Parameters	Method	Instrumentation
1	pH	Potentiometer	Digital pH meter
2	EC		Digital conductivity meter
3	TDS		Digital meter
4	Total hardness	EDTA complex	Titration
5	Chloride	Argentometry	
6	BOD	Winkler's method	
7	COD	Open refluxion	COD digester
8	Sodium	Flame Emission	Flame Photometer
9	Magnesium		
10	Calcium		
11	Phosphate	Molybdenum-blue complex	UV –Vis Spectrophotometer
12	Sulphate	Turbidimetric method	
13	Nitrate	Phenol disulponic acid

## Declaration of Competing Interest

The authors declare that they have no known competing financial interests or personal relationships which have, or could be perceived to have, influenced the work reported in this article.

## Appendix Supplementary materials

Image, application 1

Image, application 2
