# Supplementary material for: Dataset on the assessment of water quality of ground water in Kalingarayan Canal, Erode district, Tamil Nadu, India
Source: Data Brief. 2020 Aug 1;32:106112. doi: 10.1016/j.dib.2020.106112 (PMC7453106; doi:10.1016/j.dib.2020.106112)
Supplement: Supplementary file 1 [file mmc1.docx]

**APPENDIX A**- Supplementary data

**Table A1**

Parametric analysis of ground water –January 2014

| **Jan 2014** | | | | | | | | | |
| --- | --- | --- | --- | --- | --- | --- | --- | --- | --- |
| **Parameters** | **GW1** | **GW2** | **GW3** | **GW4** | **GW** | **GW6** | **GW7** | **GW8** | **GW9** |
| pH | 6.3 | 6.2 | 7.1 | 6.3 | 6.7 | 7.3 | 6.7 | 6.4 | 6.3 |
| EC | 865.0 | 1003.0 | 3480.0 | 960.0 | 1104.0 | 3145.0 | 989.0 | 725.0 | 765.0 |
| TDS | 553.6 | 641.9 | 2227.2 | 614.4 | 706.6 | 2012.8 | 633.0 | 464.0 | 489.6 |
| Chloride | 253.5 | 391.2 | 786.1 | 138.1 | 209.4 | 748.1 | 255.0 | 130.0 | 148.0 |
| Sulphate | 56.6 | 90.8 | 658.9 | 125.3 | 118.7 | 728.3 | 64.8 | 79.5 | 87.9 |
| Sodium | 97.0 | 21.9 | 561.1 | 166.0 | 196.4 | 286.3 | 118.5 | 71.9 | 98.3 |
| Calcium | 96.0 | 85.0 | 173.9 | 129.2 | 130.2 | 194.8 | 139.7 | 120.7 | 96.9 |
| Magnesium | 32.8 | 24.8 | 61.6 | 36.9 | 35.7 | 65.6 | 35.7 | 29.6 | 25.5 |
| Hardness | 374.5 | 314.2 | 687.0 | 474.1 | 471.8 | 755.6 | 495.6 | 423.1 | 346.6 |
| Nitrate | 26.1 | 31.1 | 35.1 | 30.0 | 33.5 | 33.1 | 37.5 | 38.7 | 34.6 |

Note: All the parameters are in mg/L expect pH and EC in µS/cm.

**Table A2**

Parametric analysis of ground water –February 2014

| **Feb 2014** | | | | | | | | | |
| --- | --- | --- | --- | --- | --- | --- | --- | --- | --- |
| **Parameters** | **GW1** | **GW2** | **GW3** | **GW4** | **GW** | **GW6** | **GW7** | **GW8** | **GW9** |
| pH | 6.5 | 6.6 | 7.6 | 6.8 | 7.2 | 7.8 | 7.4 | 6.8 | 6.7 |
| EC | 898.0 | 1068.0 | 3628.0 | 1075.0 | 1204.0 | 3268.0 | 1104.0 | 824.0 | 875.0 |
| TDS | 574.7 | 683.5 | 2321.9 | 688.0 | 770.6 | 2091.5 | 706.6 | 527.4 | 560.0 |
| Chloride | 264.1 | 424.3 | 808.2 | 176.6 | 239.3 | 773.4 | 276.9 | 165.0 | 183.0 |
| Sulphate | 60.7 | 95.5 | 705.1 | 137.2 | 131.4 | 766.8 | 92.7 | 91.5 | 95.8 |
| Sodium | 101.6 | 30.2 | 578.7 | 182.6 | 210.6 | 299.2 | 137.2 | 78.4 | 121.8 |
| Calcium | 99.0 | 87.0 | 176.6 | 134.1 | 133.2 | 199.8 | 143.8 | 124.5 | 101.3 |
| Magnesium | 33.6 | 24.8 | 63.1 | 38.2 | 37.1 | 67.2 | 36.7 | 31.3 | 25.9 |
| Hardness | 385.3 | 319.2 | 700.2 | 492.0 | 484.9 | 774.8 | 509.8 | 439.4 | 359.3 |
| Nitrate | 24.1 | 32.1 | 36.1 | 31.6 | 35.6 | 35.1 | 39.3 | 40.6 | 36.7 |

Note: All the parameters are in mg/L expect pH and EC in µS/cm.

**Table A3**

Parametric analysis of ground water –March 2014

| **Mar 2014** | | | | | | | | | |
| --- | --- | --- | --- | --- | --- | --- | --- | --- | --- |
| **Parameters** | **GW1** | **GW2** | **GW3** | **GW4** | **GW** | **GW6** | **GW7** | **GW8** | **GW9** |
| pH | 6.6 | 7.3 | 7.9 | 7.3 | 7.7 | 8.2 | 7.9 | 7.2 | 7.2 |
| EC | 935.0 | 1102.0 | 3654.0 | 1102.0 | 1275.0 | 3356.0 | 1187.6 | 942.0 | 961.0 |
| TDS | 598.4 | 705.3 | 2338.6 | 705.3 | 816.0 | 2147.8 | 760.1 | 602.9 | 615.0 |
| Chloride | 287.7 | 447.1 | 811.0 | 183.5 | 257.4 | 779.3 | 286.2 | 215.0 | 220.0 |
| Sulphate | 70.5 | 92.6 | 719.3 | 140.6 | 150.2 | 776.9 | 106.4 | 93.4 | 93.8 |
| Sodium | 93.0 | 36.4 | 579.2 | 188.2 | 218.0 | 316.9 | 145.0 | 93.2 | 123.5 |
| Calcium | 96.0 | 87.0 | 177.6 | 141.1 | 140.2 | 205.4 | 151.7 | 135.4 | 110.4 |
| Magnesium | 32.0 | 27.2 | 67.4 | 39.6 | 38.4 | 67.4 | 37.2 | 33.4 | 26.5 |
| Hardness | 371.2 | 329.0 | 720.3 | 515.0 | 507.8 | 789.9 | 531.9 | 475.4 | 384.6 |
| Nitrate | 24.6 | 32.6 | 34.0 | 32.5 | 36.7 | 35.6 | 40.6 | 41.8 | 37.6 |

Note: All the parameters are in mg/L expect pH and EC in µS/cm.

**Table A4**

Parametric analysis of ground water –April 2014

| **Apr 2014** | | | | | | | | | |
| --- | --- | --- | --- | --- | --- | --- | --- | --- | --- |
| **Parameters** | **GW1** | **GW2** | **GW3** | **GW4** | **GW** | **GW6** | **GW7** | **GW8** | **GW9** |
| pH | 7.6 | 7.8 | 8.4 | 7.5 | 7.7 | 8.3 | 7.7 | 6.8 | 7.2 |
| EC | 1078.9 | 1275.1 | 4051.1 | 1412.7 | 1545.2 | 3682.1 | 1374.8 | 1122.5 | 1180.0 |
| TDS | 690.5 | 816.0 | 2592.7 | 904.1 | 988.9 | 2356.6 | 879.9 | 718.4 | 755.2 |
| Chloride | 305.5 | 468.3 | 891.0 | 231.0 | 288.7 | 823.6 | 338.8 | 240.0 | 255.0 |
| Sulphate | 75.8 | 114.3 | 743.0 | 164.2 | 178.6 | 820.0 | 131.7 | 138.4 | 123.9 |
| Sodium | 105.1 | 35.7 | 638.0 | 208.7 | 238.6 | 342.5 | 166.3 | 102.8 | 128.0 |
| Calcium | 104.0 | 92.0 | 195.5 | 151.2 | 146.4 | 209.0 | 157.0 | 134.8 | 105.0 |
| Magnesium | 36.0 | 29.2 | 68.2 | 42.0 | 40.1 | 71.6 | 40.1 | 33.5 | 28.5 |
| Hardness | 407.6 | 349.7 | 768.3 | 550.1 | 530.2 | 816.2 | 556.7 | 474.5 | 379.3 |
| Nitrate | 24.9 | 33.9 | 37.9 | 35.3 | 40.1 | 36.7 | 49.7 | 42.9 | 38.7 |

Note: All the parameters are in mg/L expect pH and EC in µS/cm.

**Table A5**

Parametric analysis of ground water –May 2014

| **May 2014** | | | | | | | | | |
| --- | --- | --- | --- | --- | --- | --- | --- | --- | --- |
| **Parameters** | **GW1** | **GW2** | **GW3** | **GW4** | **GW** | **GW6** | **GW7** | **GW8** | **GW9** |
| pH | 7.8 | 7.9 | 8.4 | 7.8 | 7.9 | 8.2 | 7.5 | 7.2 | 7.1 |
| EC | 1023 | 1218 | 4002 | 1325 | 1501 | 3625 | 1435 | 1147 | 1124 |
| TDS | 654.7 | 779.5 | 2561.3 | 848.0 | 960.6 | 2320 | 918.4 | 734.1 | 719.4 |
| Chloride | 312.6 | 471.7 | 895.3 | 237.6 | 315.2 | 865.6 | 363.1 | 268.0 | 275.0 |
| Sulphate | 84.6 | 122.9 | 758.9 | 178.0 | 182.7 | 823.1 | 147.0 | 146.9 | 130.4 |
| Sodium | 108.0 | 38.0 | 642.0 | 222.8 | 247.7 | 347.5 | 173.0 | 111.7 | 135.5 |
| Calcium | 107.0 | 95.0 | 192.6 | 152.3 | 149.4 | 215.6 | 159.0 | 138.9 | 109.2 |
| Magnesium | 24.4 | 29.6 | 69.6 | 42.2 | 40.6 | 74.1 | 40.2 | 34.5 | 28.4 |
| Hardness | 367.5 | 358.9 | 766.6 | 553.6 | 540.2 | 842.9 | 562.5 | 488.7 | 389.3 |
| Nitrate | 25.3 | 34.3 | 38.3 | 35.6 | 43.5 | 37.1 | 53.8 | 46.9 | 41.9 |

Note: All the parameters are in mg/L expect pH and EC in µS/cm.

**Table A6**

Parametric analysis of ground water –June 2014

| **Jun 2014** | | | | | | | | | |
| --- | --- | --- | --- | --- | --- | --- | --- | --- | --- |
| **Parameters** | **GW1** | **GW2** | **GW3** | **GW4** | **GW** | **GW6** | **GW7** | **GW8** | **GW9** |
| pH | 7.5 | 7.7 | 8.2 | 7.6 | 7.7 | 8.0 | 7.2 | 7.0 | 6.8 |
| EC | 972 | 1175 | 3865 | 1265 | 1431 | 3524 | 1365 | 1110 | 1143 |
| TDS | 622 | 752 | 2473 | 809 | 916 | 2255 | 874 | 711 | 732 |
| Chloride | 301 | 458 | 870 | 223 | 293 | 841 | 339 | 275 | 315 |
| Sulphate | 72.1 | 116.8 | 736.6 | 171.0 | 168.1 | 800.4 | 130.9 | 133.8 | 124.8 |
| Sodium | 105.9 | 35.4 | 620.4 | 208.6 | 237.2 | 332.9 | 162.9 | 103.0 | 122.5 |
| Calcium | 105.0 | 94.0 | 186.6 | 138.0 | 143.8 | 210.4 | 156.1 | 136.1 | 102.8 |
| Magnesium | 35.2 | 28.8 | 67.4 | 41.1 | 40.0 | 72.5 | 38.5 | 33.1 | 27.8 |
| Hardness | 406.8 | 353.1 | 742.8 | 513.7 | 523.3 | 823.4 | 548.0 | 476.2 | 371.0 |
| Nitrate | 25.5 | 34.5 | 38.5 | 39.1 | 46.5 | 37.3 | 57.5 | 50.2 | 44.8 |

Note: All the parameters are in mg/L expect pH and EC in µS/cm.

**Table A7**

Parametric analysis of ground water –July 2014

| **Jul 2014** | | | | | | | | | |
| --- | --- | --- | --- | --- | --- | --- | --- | --- | --- |
| **Parameters** | **GW1** | **GW2** | **GW3** | **GW4** | **GW** | **GW6** | **GW7** | **GW8** | **GW9** |
| pH | 7.0 | 6.9 | 7.7 | 7.0 | 7.3 | 7.9 | 7.5 | 6.9 | 6.9 |
| EC | 978 | 1157 | 3871 | 1265 | 1442 | 3514 | 1342 | 1120 | 1151 |
| TDS | 625.9 | 740.5 | 2477.4 | 809.6 | 922.9 | 2249.0 | 858.9 | 717.0 | 736.9 |
| Chloride | 300.2 | 453.8 | 873.4 | 221.9 | 292.0 | 836.9 | 330.4 | 285.0 | 325.0 |
| Sulphate | 67.6 | 113.7 | 735.6 | 171.3 | 174.2 | 803.8 | 131.3 | 136.6 | 122.6 |
| Sodium | 108.4 | 34.4 | 620.4 | 208.4 | 237.2 | 333.2 | 161.3 | 102.8 | 121.6 |
| Calcium | 105.0 | 95.0 | 188.2 | 143.0 | 142.1 | 218.9 | 156.5 | 136.3 | 102.7 |
| Magnesium | 36.0 | 30.0 | 68.0 | 41.5 | 39.2 | 72.0 | 38.8 | 33.0 | 28.4 |
| Hardness | 410.1 | 360.5 | 749.1 | 527.6 | 515.8 | 842.4 | 550.2 | 476.2 | 373.3 |
| Nitrate | 25.6 | 34.4 | 38.3 | 38.7 | 45.8 | 37.1 | 56.2 | 49.1 | 43.9 |

Note: All the parameters are in mg/L expect pH and EC in µS/cm.

**Table A8**

Parametric analysis of ground water –August 2014

| **Aug 2014** | | | | | | | | | |
| --- | --- | --- | --- | --- | --- | --- | --- | --- | --- |
| **Parameters** | **GW1** | **GW2** | **GW3** | **GW4** | **GW** | **GW6** | **GW7** | **GW8** | **GW9** |
| pH | 7.0 | 6.9 | 7.6 | 6.9 | 7.3 | 7.8 | 7.5 | 7.0 | 7.0 |
| EC | 1067.7 | 1262.3 | 3960.8 | 1388.8 | 1519.4 | 3658.4 | 1354.0 | 1099.3 | 1134.3 |
| TDS | 683.3 | 807.9 | 2534.9 | 888.8 | 972.4 | 2341.4 | 866.6 | 703.6 | 726.0 |
| Chloride | 299.3 | 449.4 | 872.0 | 215.5 | 293.0 | 838.2 | 333.6 | 275.0 | 315.0 |
| Sulphate | 66.0 | 113.4 | 728.8 | 170.6 | 175.5 | 792.6 | 131.6 | 135.5 | 116.4 |
| Sodium | 106.0 | 34.4 | 615.5 | 205.1 | 234.2 | 328.7 | 158.6 | 100.6 | 120.5 |
| Calcium | 105.0 | 93.0 | 185.9 | 141.3 | 140.4 | 214.9 | 154.9 | 134.1 | 102.6 |
| Magnesium | 36.0 | 30.0 | 67.4 | 41.0 | 38.7 | 70.9 | 38.2 | 32.8 | 28.3 |
| Hardness | 410.1 | 355.5 | 740.9 | 521.6 | 509.7 | 827.8 | 544.0 | 469.6 | 372.4 |
| Nitrate | 25.3 | 34.1 | 38.0 | 38.3 | 45.1 | 36.8 | 55.0 | 48.1 | 43.0 |

Note: All the parameters are in mg/L expect pH and EC in µS/cm.

**Table A9**

Parametric analysis of ground water – September 2014

| **Sep 2014** | | | | | | | | | |
| --- | --- | --- | --- | --- | --- | --- | --- | --- | --- |
| **Parameters** | **GW1** | **GW2** | **GW3** | **GW4** | **GW** | **GW6** | **GW7** | **GW8** | **GW9** |
| pH | 6.5 | 6.4 | 7.2 | 6.5 | 6.8 | 7.3 | 7.0 | 6.5 | 6.5 |
| EC | 897 | 1075 | 3629 | 1095 | 1214 | 3265 | 1164 | 1035 | 1065 |
| TDS | 574.1 | 688.0 | 2322.6 | 700.8 | 777.0 | 2089.6 | 745.1 | 662.4 | 681.6 |
| Chloride | 277.6 | 417.2 | 819.7 | 176.1 | 248.3 | 791.8 | 288.7 | 280.0 | 310.0 |
| Sulphate | 58.5 | 105.7 | 689.0 | 144.0 | 148.8 | 746.8 | 92.2 | 104.7 | 104.6 |
| Sodium | 96.4 | 26.1 | 581.2 | 184.6 | 210.2 | 304.9 | 138.4 | 84.5 | 109.3 |
| Calcium | 99.0 | 88.0 | 176.2 | 133.9 | 132.9 | 204.2 | 147.3 | 127.1 | 97.3 |
| Magnesium | 30.8 | 29.2 | 64.7 | 38.9 | 36.6 | 67.0 | 36.2 | 31.2 | 26.8 |
| Hardness | 373.8 | 339.7 | 705.9 | 494.2 | 482.3 | 785.2 | 516.8 | 445.7 | 352.9 |
| Nitrate | 15.1 | 20.1 | 31.1 | 38.3 | 15.1 | 22.1 | 55.0 | 48.1 | 43.0 |

Note: All the parameters are in mg/L expect pH and EC in µS/cm.

**Table A10**

Parametric analysis of ground water –October 2014

| **Oct 2014** | | | | | | | | | |
| --- | --- | --- | --- | --- | --- | --- | --- | --- | --- |
| **Parameters** | **GW1** | **GW2** | **GW3** | **GW4** | **GW** | **GW6** | **GW7** | **GW8** | **GW9** |
| pH | 6.1 | 6.0 | 6.8 | 6.0 | 6.4 | 6.9 | 6.6 | 6.1 | 6.0 |
| EC | 851.0 | 1003.0 | 3415.0 | 952.0 | 1095.0 | 3104.0 | 992.0 | 987.0 | 1035.0 |
| TDS | 544.6 | 641.9 | 2185.6 | 609.3 | 700.8 | 1986.6 | 634.9 | 631.7 | 662.4 |
| Chloride | 261.8 | 396.2 | 773.6 | 142.2 | 212.3 | 749.6 | 248.0 | 287.0 | 318.0 |
| Sulphate | 53.2 | 98.4 | 648.3 | 126.2 | 125.2 | 711.6 | 62.2 | 79.0 | 87.1 |
| Sodium | 90.1 | 22.9 | 555.2 | 169.0 | 192.0 | 284.3 | 117.0 | 71.1 | 97.6 |
| Calcium | 95.0 | 83.0 | 169.9 | 126.7 | 126.7 | 192.0 | 138.2 | 121.0 | 92.2 |
| Magnesium | 32.0 | 27.6 | 61.6 | 36.9 | 34.9 | 64.5 | 34.2 | 29.6 | 25.3 |
| Hardness | 368.7 | 320.7 | 677.5 | 467.9 | 460.1 | 744.5 | 485.7 | 423.6 | 334.3 |
| Nitrate | 6.1 | 6.0 | 6.8 | 6.0 | 6.4 | 6.9 | 6.6 | 6.1 | 6.0 |

Note: All the parameters are in mg/L expect pH and EC in µS/cm.

**Table A11**

Parametric analysis of ground water –November 2014

| **Nov 2014** | | | | | | | | | |
| --- | --- | --- | --- | --- | --- | --- | --- | --- | --- |
| **Parameters** | **GW1** | **GW2** | **GW3** | **GW4** | **GW** | **GW6** | **GW7** | **GW8** | **GW9** |
| pH | 6.0 | 5.9 | 6.6 | 5.9 | 6.3 | 6.7 | 6.5 | 6.0 | 5.9 |
| EC | 825.0 | 985.0 | 3345.0 | 1035.0 | 1045.0 | 3022.0 | 933.0 | 899 | 965.0 |
| TDS | 528.0 | 630.4 | 2140.8 | 662.4 | 668.8 | 1934.1 | 597.1 | 575.4 | 617.6 |
| Chloride | 250.5 | 383.8 | 755.6 | 225.0 | 198.5 | 727.0 | 242.3 | 280.0 | 310.0 |
| Sulphate | 50.2 | 94.0 | 631.9 | 116.7 | 115.8 | 694.7 | 47.1 | 68.1 | 80.1 |
| Sodium | 86.8 | 20.7 | 539.5 | 160.1 | 182.9 | 268.2 | 113.4 | 64.9 | 89.2 |
| Calcium | 93.0 | 81.0 | 163.7 | 123.8 | 124.7 | 187.5 | 138.0 | 114.2 | 90.4 |
| Magnesium | 31.6 | 27.2 | 60.0 | 35.8 | 34.3 | 63.4 | 33.9 | 28.2 | 24.8 |
| Hardness | 362.1 | 314.0 | 655.3 | 456.2 | 452.3 | 728.8 | 484.1 | 401.1 | 327.6 |
| Nitrate | 21.1 | 23.1 | 28.1 | 21.1 | 18.1 | 31.1 | 32.1 | 33.1 | 24.1 |

Note: All the parameters are in mg/L expect pH and EC in µS/cm.

**Table A12**

Parametric analysis of ground water –December 2014

| **Dec 2014** | | | | | | | | | |
| --- | --- | --- | --- | --- | --- | --- | --- | --- | --- |
| **Parameters** | **GW1** | **GW2** | **GW3** | **GW4** | **GW** | **GW6** | **GW7** | **GW8** | **GW9** |
| pH | 6.1 | 6.1 | 6.9 | 6.1 | 6.5 | 7.0 | 6.7 | 6.2 | 6.1 |
| EC | 906.4 | 1071.6 | 3534.8 | 1062.0 | 1199.5 | 3231.2 | 1010.9 | 830.7 | 918.5 |
| TDS | 580.1 | 685.8 | 2262.3 | 679.7 | 767.7 | 2068.0 | 647.0 | 531.7 | 587.8 |
| Chloride | 255.7 | 387.5 | 783.2 | 235.0 | 209.4 | 748.8 | 245.0 | 281.0 | 315.0 |
| Sulphate | 54.4 | 96.5 | 654.1 | 125.8 | 119.1 | 716.2 | 57.0 | 80.2 | 88.9 |
| Sodium | 92.1 | 24.3 | 560.5 | 168.0 | 195.7 | 280.0 | 118.3 | 71.5 | 92.7 |
| Calcium | 95.0 | 85.0 | 169.0 | 125.1 | 127.0 | 193.9 | 140.4 | 117.5 | 92.6 |
| Magnesium | 32.4 | 27.6 | 61.9 | 33.2 | 31.6 | 65.3 | 34.8 | 29.0 | 25.2 |
| Hardness | 370.3 | 325.7 | 676.3 | 448.9 | 446.9 | 752.5 | 493.5 | 412.7 | 335.0 |
| Nitrate | 28.1 | 31.1 | 36.1 | 28.1 | 31.1 | 34.1 | 34.6 | 35.6 | 32.1 |

Note: All the parameters are in mg/L expect pH and EC in µS/cm.

**Table A13**

Parametric analysis of ground water –January 2015

| **Jan 2015** | | | | | | | | | |
| --- | --- | --- | --- | --- | --- | --- | --- | --- | --- |
| **Parameters** | **GW1** | **GW2** | **GW3** | **GW4** | **GW** | **GW6** | **GW7** | **GW8** | **GW9** |
| pH | 6.2 | 6.2 | 7.0 | 6.2 | 6.6 | 7.1 | 6.8 | 6.3 | 6.2 |
| EC | 921.4 | 1110.6 | 3573.8 | 1165.0 | 1198.0 | 3215.0 | 1049.9 | 1045.0 | 1078.0 |
| TDS | 589.7 | 710.8 | 2287.3 | 745.6 | 766.7 | 2057.6 | 671.9 | 668.8 | 689.9 |
| Chloride | 280.7 | 412.5 | 808.2 | 260.0 | 234.4 | 773.8 | 270.0 | 306.0 | 340.0 |
| Sulphate | 58.4 | 100.5 | 658.1 | 129.8 | 123.1 | 720.2 | 61.0 | 84.2 | 92.9 |
| Sodium | 102.1 | 34.3 | 570.5 | 178.0 | 205.7 | 290.0 | 128.3 | 81.5 | 102.7 |
| Calcium | 101.0 | 91.0 | 175.0 | 131.1 | 133.0 | 199.9 | 146.4 | 123.5 | 98.6 |
| Magnesium | 39.4 | 34.6 | 68.9 | 40.2 | 38.6 | 72.3 | 41.8 | 36.0 | 32.2 |
| Hardness | 414.0 | 369.4 | 720.0 | 492.6 | 490.6 | 796.2 | 537.2 | 456.4 | 378.7 |
| Nitrate | 34.1 | 37.1 | 42.1 | 34.1 | 37.1 | 40.1 | 40.6 | 41.6 | 38.1 |

Note: All the parameters are in mg/L expect pH and EC in µS/cm.

**Table A14**

Parametric analysis of ground water –February 2015

| **Feb 2015** | | | | | | | | | |
| --- | --- | --- | --- | --- | --- | --- | --- | --- | --- |
| **Parameters** | **GW1** | **GW2** | **GW3** | **GW4** | **GW** | **GW6** | **GW7** | **GW8** | **GW9** |
| pH | 6.8 | 6.9 | 8.0 | 7.1 | 7.6 | 8.2 | 7.8 | 7.1 | 7.0 |
| EC | 942.9 | 1121.4 | 3809.4 | 1128.8 | 1264.2 | 3431.4 | 1159.2 | 865.2 | 918.8 |
| TDS | 603.5 | 717.7 | 2438.0 | 722.4 | 809.1 | 2196.1 | 741.9 | 553.7 | 588.0 |
| Chloride | 277.3 | 445.5 | 848.6 | 185.4 | 251.2 | 812.1 | 290.8 | 173.3 | 192.2 |
| Sulphate | 63.8 | 100.2 | 740.3 | 144.1 | 138.0 | 805.2 | 97.3 | 96.1 | 100.6 |
| Sodium | 106.7 | 31.7 | 607.6 | 191.7 | 221.1 | 314.1 | 144.1 | 82.3 | 127.9 |
| Calcium | 104.0 | 91.4 | 185.4 | 140.8 | 139.8 | 209.7 | 151.0 | 130.7 | 106.4 |
| Magnesium | 35.3 | 26.0 | 66.3 | 40.1 | 38.9 | 70.5 | 38.5 | 32.8 | 27.2 |
| Hardness | 404.5 | 335.1 | 735.3 | 516.6 | 509.1 | 813.5 | 535.3 | 461.4 | 377.3 |
| Nitrate | 34.4 | 40.3 | 42.3 | 36.4 | 40.3 | 43.3 | 37.4 | 36.4 | 33.4 |

Note: All the parameters are in mg/L expect pH and EC in µS/cm.

**Table A15**

Parametric analysis of ground water –March 2015

| **Mar 2015** | | | | | | | | | |
| --- | --- | --- | --- | --- | --- | --- | --- | --- | --- |
| **Parameters** | **GW1** | **GW2** | **GW3** | **GW4** | **GW** | **GW6** | **GW7** | **GW8** | **GW9** |
| pH | 7.0 | 7.8 | 8.4 | 7.8 | 8.2 | 8.8 | 8.4 | 7.7 | 7.7 |
| EC | 995.8 | 1173.6 | 3891.5 | 1173.6 | 1357.9 | 3574.1 | 1264.8 | 1003.2 | 1023.5 |
| TDS | 637.3 | 751.1 | 2490.6 | 751.1 | 869.0 | 2287.4 | 809.5 | 642.1 | 655.0 |
| Chloride | 306.4 | 476.1 | 863.7 | 195.4 | 274.1 | 830.0 | 304.8 | 229.0 | 234.3 |
| Sulphate | 75.1 | 98.6 | 766.1 | 149.7 | 160.0 | 827.4 | 113.3 | 99.5 | 99.9 |
| Sodium | 99.0 | 38.7 | 616.8 | 200.4 | 232.1 | 337.5 | 154.4 | 99.2 | 131.5 |
| Calcium | 102.2 | 92.7 | 189.1 | 150.3 | 149.3 | 218.8 | 161.5 | 144.2 | 117.6 |
| Magnesium | 34.1 | 29.0 | 71.8 | 42.1 | 40.9 | 71.8 | 39.7 | 35.6 | 28.2 |
| Hardness | 395.3 | 350.4 | 767.1 | 548.4 | 540.8 | 841.3 | 566.5 | 506.3 | 409.6 |
| Nitrate | 35.1 | 41.0 | 42.9 | 38.1 | 42.0 | 46.8 | 37.1 | 37.1 | 34.2 |

Note: All the parameters are in mg/L expect pH and EC in µS/cm.

**Table A16**

Parametric analysis of ground water – April 2015

| **Apr 2015** | | | | | | | | | |
| --- | --- | --- | --- | --- | --- | --- | --- | --- | --- |
| **Parameters** | **GW1** | **GW2** | **GW3** | **GW4** | **GW** | **GW6** | **GW7** | **GW8** | **GW9** |
| pH | 8.1 | 8.3 | 8.9 | 8.0 | 8.2 | 8.8 | 8.2 | 7.2 | 7.6 |
| EC | 1143.6 | 1351.6 | 4294.2 | 1345.0 | 1637.9 | 3903.1 | 1457.3 | 1189.9 | 1125.0 |
| TDS | 731.9 | 865.0 | 2748.3 | 860.8 | 1048.2 | 2498.0 | 932.7 | 761.5 | 720.0 |
| Chloride | 323.9 | 496.4 | 944.4 | 244.8 | 306.1 | 873.0 | 359.1 | 254.4 | 270.3 |
| Sulphate | 80.4 | 121.2 | 787.5 | 174.1 | 189.4 | 869.2 | 139.6 | 146.7 | 131.3 |
| Sodium | 111.4 | 37.9 | 676.3 | 221.2 | 252.9 | 363.1 | 176.3 | 109.0 | 135.7 |
| Calcium | 110.2 | 97.5 | 207.2 | 160.3 | 155.2 | 221.5 | 166.4 | 142.9 | 111.3 |
| Magnesium | 38.2 | 31.0 | 72.3 | 44.5 | 42.5 | 75.9 | 42.5 | 35.5 | 30.2 |
| Hardness | 432.1 | 370.7 | 814.4 | 583.1 | 562.0 | 865.2 | 590.1 | 502.9 | 402.0 |
| Nitrate | 26.4 | 35.9 | 40.2 | 37.4 | 42.5 | 38.9 | 52.7 | 45.5 | 41.0 |

Note: All the parameters are in mg/L expect pH and EC in µS/cm.

**Table A17**

Parametric analysis of ground water – May 2015

| **May 2015** | | | | | | | | | |
| --- | --- | --- | --- | --- | --- | --- | --- | --- | --- |
| **Parameters** | **GW1** | **GW2** | **GW3** | **GW4** | **GW** | **GW6** | **GW7** | **GW8** | **GW9** |
| pH | 8.1 | 8.3 | 8.8 | 8.1 | 8.2 | 8.6 | 7.8 | 7.5 | 7.4 |
| EC | 1058.0 | 1525.0 | 4162.1 | 1378.0 | 1561.0 | 3770.0 | 1492.4 | 1192.9 | 1169.0 |
| TDS | 677.1 | 976.0 | 2663.7 | 881.9 | 999.1 | 2412.8 | 955.1 | 763.4 | 748.1 |
| Chloride | 325.1 | 490.5 | 931.1 | 247.1 | 327.8 | 900.2 | 377.6 | 278.7 | 286.0 |
| Sulphate | 87.9 | 127.8 | 789.2 | 185.1 | 190.0 | 856.0 | 152.9 | 152.8 | 135.6 |
| Sodium | 112.3 | 39.6 | 667.7 | 231.7 | 257.6 | 361.4 | 179.9 | 116.1 | 140.9 |
| Calcium | 111.3 | 98.8 | 200.3 | 158.4 | 155.4 | 224.2 | 165.4 | 144.5 | 113.6 |
| Magnesium | 25.4 | 30.8 | 72.3 | 43.8 | 42.2 | 77.1 | 41.8 | 35.9 | 29.5 |
| Hardness | 382.2 | 373.2 | 797.2 | 575.8 | 561.8 | 876.6 | 585.0 | 508.2 | 404.9 |
| Nitrate | 26.3 | 35.6 | 39.8 | 37.1 | 45.3 | 38.5 | 56.0 | 48.8 | 43.6 |

Note: All the parameters are in mg/L expect pH and EC in µS/cm.

**Table A18**

Parametric analysis of ground water – June 2015

| **Jun 2015** | | | | | | | | | |
| --- | --- | --- | --- | --- | --- | --- | --- | --- | --- |
| **Parameters** | **GW1** | **GW2** | **GW3** | **GW4** | **GW** | **GW6** | **GW7** | **GW8** | **GW9** |
| pH | 8.1 | 8.4 | 8.9 | 8.3 | 8.4 | 8.7 | 7.8 | 7.6 | 7.4 |
| EC | 1054.6 | 1274.9 | 4193.5 | 1372.5 | 1552.0 | 3823.5 | 1481.0 | 1204.9 | 1240.7 |
| TDS | 675.0 | 815.9 | 2683.9 | 878.4 | 993.3 | 2447.1 | 947.9 | 771.1 | 794.0 |
| Chloride | 326.6 | 497.0 | 943.8 | 242.3 | 318.1 | 912.8 | 367.2 | 298.4 | 341.8 |
| Sulphate | 78.2 | 126.8 | 799.2 | 185.5 | 182.4 | 868.4 | 142.0 | 145.2 | 135.4 |
| Sodium | 114.9 | 38.4 | 673.2 | 226.4 | 257.4 | 361.2 | 176.8 | 111.7 | 132.9 |
| Calcium | 113.9 | 102.0 | 202.5 | 149.8 | 156.0 | 228.3 | 169.4 | 147.7 | 111.6 |
| Magnesium | 38.2 | 31.2 | 73.1 | 44.6 | 43.4 | 78.7 | 41.7 | 35.9 | 30.2 |
| Hardness | 441.4 | 383.1 | 806.0 | 557.4 | 567.8 | 893.4 | 594.6 | 516.6 | 402.5 |
| Nitrate | 27.7 | 37.4 | 41.8 | 42.4 | 50.5 | 40.5 | 62.4 | 54.4 | 48.7 |

Note: All the parameters are in mg/L expect pH and EC in µS/cm.

**Table A19**

Parametric analysis of ground water – July 2015

| **Jul 2015** | | | | | | | | | |
| --- | --- | --- | --- | --- | --- | --- | --- | --- | --- |
| **Parameters** | **GW1** | **GW2** | **GW3** | **GW4** | **GW** | **GW6** | **GW7** | **GW8** | **GW9** |
| pH | 7.5 | 7.4 | 8.2 | 7.5 | 7.8 | 8.4 | 8.0 | 7.4 | 7.4 |
| EC | 1046.5 | 1238.0 | 4142.0 | 1353.6 | 1542.9 | 3760.0 | 1435.9 | 1198.8 | 1232.0 |
| TDS | 669.7 | 792.3 | 2650.9 | 866.3 | 987.5 | 2406.4 | 919.0 | 767.2 | 788.5 |
| Chloride | 321.2 | 485.6 | 934.5 | 237.4 | 312.4 | 895.5 | 353.5 | 305.0 | 347.8 |
| Sulphate | 72.4 | 121.7 | 787.1 | 183.3 | 186.4 | 860.1 | 140.5 | 146.2 | 131.2 |
| Sodium | 115.9 | 36.9 | 663.9 | 222.9 | 253.8 | 356.6 | 172.6 | 110.0 | 130.1 |
| Calcium | 112.4 | 101.7 | 201.3 | 153.1 | 152.0 | 234.2 | 167.4 | 145.9 | 109.9 |
| Magnesium | 38.5 | 32.1 | 72.7 | 44.4 | 41.9 | 77.0 | 41.5 | 35.3 | 30.4 |
| Hardness | 438.8 | 385.7 | 801.5 | 564.6 | 551.9 | 901.4 | 588.7 | 509.5 | 399.4 |
| Nitrate | 27.4 | 36.8 | 41.0 | 41.4 | 49.0 | 39.7 | 60.2 | 52.6 | 47.0 |

Note: All the parameters are in mg/L expect pH and EC in µS/cm.

**Table A20**

Parametric analysis of ground water – August 2015

| **Aug 2015** | | | | | | | | | |
| --- | --- | --- | --- | --- | --- | --- | --- | --- | --- |
| **Parameters** | **GW1** | **GW2** | **GW3** | **GW4** | **GW** | **GW6** | **GW7** | **GW8** | **GW9** |
| pH | 7.5 | 7.4 | 8.2 | 7.4 | 7.8 | 8.4 | 8.0 | 7.5 | 7.5 |
| EC | 1045.0 | 1253.0 | 4147.0 | 1325.0 | 1985.0 | 3923.7 | 1452.2 | 1179.0 | 1216.6 |
| TDS | 668.8 | 801.9 | 2654.1 | 848.0 | 1270.4 | 2511.2 | 929.4 | 754.6 | 778.6 |
| Chloride | 321.0 | 481.9 | 935.3 | 231.1 | 614.0 | 898.9 | 357.8 | 294.9 | 337.8 |
| Sulphate | 70.8 | 121.7 | 781.6 | 183.0 | 188.2 | 850.1 | 141.2 | 145.3 | 124.8 |
| Sodium | 113.7 | 36.9 | 660.1 | 220.0 | 251.1 | 352.5 | 170.1 | 107.9 | 129.3 |
| Calcium | 112.6 | 99.7 | 199.3 | 151.6 | 150.5 | 230.5 | 166.1 | 143.8 | 110.0 |
| Magnesium | 38.6 | 32.2 | 72.3 | 44.0 | 41.5 | 76.0 | 41.0 | 35.2 | 30.3 |
| Hardness | 439.8 | 381.3 | 794.6 | 559.4 | 546.6 | 887.8 | 583.4 | 503.7 | 399.4 |
| Nitrate | 27.1 | 36.6 | 40.8 | 41.0 | 48.3 | 39.5 | 59.0 | 51.6 | 46.1 |

Note: All the parameters are in mg/L expect pH and EC in µS/cm.

**Table A21**

Parametric analysis of ground water – September 2015

| **Sep 2015** | | | | | | | | | |
| --- | --- | --- | --- | --- | --- | --- | --- | --- | --- |
| **Parameters** | **GW1** | **GW2** | **GW3** | **GW4** | **GW** | **GW6** | **GW7** | **GW8** | **GW9** |
| pH | 6.9 | 6.8 | 7.7 | 6.9 | 7.3 | 7.8 | 7.5 | 7.0 | 6.9 |
| EC | 953.1 | 1142.2 | 3855.8 | 1163.4 | 1289.9 | 3469.1 | 1237.0 | 1099.7 | 1131.6 |
| TDS | 610.0 | 731.0 | 2467.7 | 744.6 | 825.5 | 2220.2 | 791.7 | 703.8 | 724.2 |
| Chloride | 295.0 | 443.3 | 870.9 | 187.1 | 263.8 | 841.3 | 306.8 | 297.5 | 329.4 |
| Sulphate | 62.1 | 112.3 | 732.1 | 153.0 | 158.1 | 793.5 | 98.0 | 111.2 | 111.2 |
| Sodium | 102.4 | 27.7 | 617.5 | 196.2 | 223.3 | 324.0 | 147.1 | 89.8 | 116.1 |
| Calcium | 105.2 | 93.5 | 187.2 | 142.2 | 141.2 | 216.9 | 156.5 | 135.1 | 103.3 |
| Magnesium | 32.7 | 31.0 | 68.8 | 41.3 | 38.9 | 71.2 | 38.5 | 33.2 | 28.4 |
| Hardness | 397.1 | 361.0 | 750.0 | 525.0 | 512.4 | 834.3 | 549.1 | 473.6 | 375.0 |
| Nitrate | 16.0 | 21.4 | 33.0 | 40.7 | 16.0 | 23.5 | 58.4 | 51.1 | 45.6 |

Note: All the parameters are in mg/L expect pH and EC in µS/cm.

**Table A22**

Parametric analysis of ground water – October 2015

| **Oct 2015** | | | | | | | | | |
| --- | --- | --- | --- | --- | --- | --- | --- | --- | --- |
| **Parameters** | **GW1** | **GW2** | **GW3** | **GW4** | **GW** | **GW6** | **GW7** | **GW8** | **GW9** |
| pH | 6.6 | 6.5 | 7.4 | 6.5 | 7.0 | 7.5 | 7.2 | 6.6 | 6.5 |
| EC | 921.2 | 1085.7 | 3696.7 | 1030.5 | 1185.3 | 3360.1 | 1073.8 | 1068.4 | 1120.4 |
| TDS | 589.6 | 694.9 | 2365.9 | 659.5 | 758.6 | 2150.5 | 687.3 | 683.8 | 717.0 |
| Chloride | 283.4 | 428.9 | 837.4 | 153.9 | 229.8 | 811.4 | 268.5 | 310.7 | 344.2 |
| Sulphate | 57.6 | 106.5 | 701.8 | 136.6 | 135.6 | 770.4 | 67.3 | 85.5 | 94.3 |
| Sodium | 97.6 | 24.8 | 601.0 | 182.9 | 207.9 | 307.7 | 126.7 | 76.9 | 105.6 |
| Calcium | 102.8 | 89.8 | 183.9 | 137.2 | 137.2 | 207.8 | 149.6 | 130.9 | 99.8 |
| Magnesium | 34.6 | 29.9 | 66.7 | 39.9 | 37.8 | 69.8 | 37.0 | 32.0 | 27.4 |
| Hardness | 399.1 | 347.1 | 733.4 | 506.5 | 498.0 | 805.9 | 525.8 | 458.6 | 361.9 |
| Nitrate | 19.6 | 22.8 | 26.1 | 17.4 | 19.6 | 25.0 | 48.8 | 49.9 | 46.7 |

Note: All the parameters are in mg/L expect pH and EC in µS/cm.

**Table A23**

Parametric analysis of ground water – November 2015

| **Nov 2015** | | | | | | | | | |
| --- | --- | --- | --- | --- | --- | --- | --- | --- | --- |
| **Parameters** | **GW1** | **GW2** | **GW3** | **GW4** | **GW** | **GW6** | **GW7** | **GW8** | **GW9** |
| pH | 6.5 | 6.4 | 7.2 | 6.4 | 6.8 | 7.3 | 7.0 | 6.5 | 6.4 |
| EC | 891.0 | 1063.8 | 3612.6 | 1117.8 | 1128.6 | 3263.8 | 1007.6 | 970.9 | 1042.2 |
| TDS | 570.2 | 680.8 | 2312.1 | 715.4 | 722.3 | 2088.8 | 644.9 | 621.4 | 667.0 |
| Chloride | 270.6 | 414.5 | 816.0 | 243.0 | 214.4 | 785.2 | 261.7 | 302.4 | 334.8 |
| Sulphate | 54.2 | 101.5 | 682.4 | 126.1 | 125.0 | 750.3 | 50.9 | 73.6 | 86.5 |
| Sodium | 93.8 | 22.3 | 582.7 | 172.9 | 197.6 | 289.6 | 122.5 | 70.1 | 96.3 |
| Calcium | 100.4 | 87.5 | 176.8 | 133.7 | 134.7 | 202.5 | 149.1 | 123.4 | 97.7 |
| Magnesium | 34.1 | 29.4 | 64.8 | 38.7 | 37.0 | 68.5 | 36.6 | 30.4 | 26.7 |
| Hardness | 391.0 | 339.1 | 707.7 | 492.7 | 488.5 | 787.1 | 522.8 | 433.2 | 353.8 |
| Nitrate | 22.8 | 24.9 | 30.3 | 22.8 | 19.5 | 33.6 | 34.7 | 35.7 | 26.0 |

Note: All the parameters are in mg/L expect pH and EC in µS/cm.

**Table A24**

Parametric analysis of ground water – December 2015

| **Dec 2015** | | | | | | | | | |
| --- | --- | --- | --- | --- | --- | --- | --- | --- | --- |
| **Parameters** | **GW1** | **GW2** | **GW3** | **GW4** | **GW** | **GW6** | **GW7** | **GW8** | **GW9** |
| pH | 6.6 | 6.6 | 7.4 | 6.6 | 7.0 | 7.5 | 7.2 | 6.7 | 6.6 |
| EC | 974.9 | 1152.6 | 3802.1 | 1142.2 | 1290.2 | 3475.5 | 1087.3 | 1045.0 | 1095.0 |
| TDS | 623.9 | 737.7 | 2433.3 | 731.0 | 825.7 | 2224.3 | 695.9 | 668.8 | 700.8 |
| Chloride | 275.0 | 416.8 | 842.4 | 252.8 | 225.2 | 805.4 | 263.5 | 302.2 | 338.8 |
| Sulphate | 58.5 | 103.7 | 703.6 | 135.3 | 128.1 | 770.3 | 61.3 | 86.3 | 95.6 |
| Sodium | 99.1 | 26.2 | 602.9 | 180.7 | 210.4 | 301.2 | 127.2 | 76.9 | 99.7 |
| Calcium | 102.2 | 91.4 | 181.8 | 134.6 | 136.6 | 208.5 | 151.0 | 126.3 | 99.6 |
| Magnesium | 34.8 | 29.7 | 66.6 | 35.7 | 33.9 | 70.3 | 37.4 | 31.2 | 27.1 |
| Hardness | 398.3 | 350.3 | 727.4 | 482.8 | 480.7 | 809.4 | 530.8 | 443.9 | 360.3 |
| Nitrate | 30.2 | 33.5 | 38.8 | 30.2 | 33.5 | 36.7 | 37.2 | 38.3 | 34.5 |

Note: All the parameters are in mg/L expect pH and EC in µS/cm.

**Table A25**

Parametric analysis of ground water – January 2016

| **Jan 2016** | | | | | | | | | |
| --- | --- | --- | --- | --- | --- | --- | --- | --- | --- |
| **Parameters** | **GW1** | **GW2** | **GW3** | **GW4** | **GW** | **GW6** | **GW7** | **GW8** | **GW9** |
| pH | 6.6 | 6.5 | 7.5 | 7.1 | 7.6 | 7.7 | 7.8 | 7.3 | 7.1 |
| EC | 1075 | 1205 | 3709 | 1295 | 1302 | 3315 | 1109 | 1135 | 1190 |
| TDS | 688.0 | 771.2 | 2373.8 | 828.8 | 833.3 | 2121.6 | 709.8 | 726.4 | 761.8 |
| Chloride | 326.0 | 471.0 | 834.0 | 274.8 | 244.8 | 794.0 | 286.4 | 328.5 | 368.3 |
| Sulphate | 84.0 | 120.0 | 697.0 | 148.8 | 139.2 | 770.0 | 66.6 | 93.8 | 104.0 |
| Sodium | 116.0 | 37.0 | 594.0 | 196.4 | 228.7 | 310.0 | 138.3 | 83.6 | 108.4 |
| Calcium | 101.0 | 90.0 | 183.0 | 146.3 | 148.5 | 205.0 | 164.1 | 137.3 | 108.3 |
| Magnesium | 51.6 | 44.4 | 91.8 | 58.2 | 55.3 | 97.8 | 61.0 | 50.9 | 44.2 |
| Hardness | 464.1 | 407.0 | 833.9 | 604.4 | 598.2 | 913.3 | 660.3 | 552.1 | 452.1 |
| Nitrate | 29.0 | 34.0 | 38.0 | 32.9 | 36.4 | 36.0 | 40.4 | 41.6 | 37.5 |

Note: All the parameters are in mg/L expect pH and EC in µS/cm.

**Table A26**

Parametric analysis of ground water – February 2016

| **Feb 2016** | | | | | | | | | |
| --- | --- | --- | --- | --- | --- | --- | --- | --- | --- |
| **Parameters** | **GW1** | **GW2** | **GW3** | **GW4** | **GW** | **GW6** | **GW7** | **GW8** | **GW9** |
| pH | 6.7 | 6.8 | 7.9 | 7.5 | 8.0 | 8.1 | 8.2 | 7.6 | 7.5 |
| EC | 1087 | 1265 | 3802 | 1360 | 1380 | 3409 | 1159 | 1186 | 1256 |
| TDS | 695.7 | 809.6 | 2433.3 | 870.2 | 883.3 | 2,181.8 | 741.7 | 759.1 | 803.7 |
| Chloride | 330.0 | 496.0 | 842.0 | 288.5 | 259.5 | 806.0 | 299.3 | 343.3 | 388.5 |
| Sulphate | 86.0 | 122.0 | 733.0 | 156.2 | 147.6 | 797.0 | 69.6 | 98.0 | 109.7 |
| Sodium | 118.0 | 44.0 | 602.0 | 206.2 | 242.5 | 316.0 | 144.5 | 87.4 | 114.3 |
| Calcium | 102.0 | 91.0 | 183.0 | 153.6 | 157.4 | 207.0 | 171.5 | 143.5 | 114.3 |
| Magnesium | 52.2 | 45.0 | 92.7 | 61.1 | 58.7 | 98.6 | 63.7 | 53.2 | 46.6 |
| Hardness | 469.0 | 412.0 | 837.4 | 634.6 | 634.1 | 921.8 | 690.0 | 576.9 | 476.9 |
| Nitrate | 27.0 | 35.0 | 39.0 | 34.5 | 38.5 | 38.0 | 42.2 | 43.5 | 39.6 |

Note: All the parameters are in mg/L expect pH and EC in µS/cm.

**Table A27**

Parametric analysis of ground water – March 2016

| **Mar 2016** | | | | | | | | | |
| --- | --- | --- | --- | --- | --- | --- | --- | --- | --- |
| **Parameters** | **GW1** | **GW2** | **GW3** | **GW4** | **GW** | **GW6** | **GW7** | **GW8** | **GW9** |
| pH | 6.9 | 7.6 | 8.4 | 7.7 | 8.3 | 8.6 | 8.4 | 7.8 | 7.7 |
| EC | 1245 | 1414 | 3999 | 1394 | 1419 | 3502 | 1194 | 1218 | 1286 |
| TDS | 796.8 | 905.0 | 2559.4 | 892.0 | 908.0 | 2,241.3 | 764.0 | 779.6 | 823.0 |
| Chloride | 357.0 | 523.0 | 850.0 | 295.7 | 266.8 | 817.0 | 308.3 | 352.6 | 397.9 |
| Sulphate | 97.0 | 120.0 | 752.0 | 168.0 | 151.7 | 812.0 | 71.7 | 100.6 | 112.3 |
| Sodium | 110.0 | 51.0 | 606.0 | 211.3 | 249.3 | 337.0 | 148.9 | 89.7 | 117.1 |
| Calcium | 101.0 | 92.0 | 185.0 | 157.4 | 161.8 | 214.0 | 176.7 | 147.4 | 117.0 |
| Magnesium | 50.4 | 42.6 | 99.5 | 62.7 | 60.3 | 99.5 | 65.6 | 54.6 | 47.8 |
| Hardness | 459.1 | 404.7 | 870.2 | 650.5 | 651.8 | 942.7 | 710.7 | 592.5 | 488.4 |
| Nitrate | 27.5 | 35.5 | 39.5 | 35.4 | 39.6 | 38.5 | 43.5 | 44.7 | 40.5 |

Note: All the parameters are in mg/L expect pH and EC in µS/cm.

**Table A28**

Parametric analysis of ground water – April 2016

| **Apr 2016** | | | | | | | | | |
| --- | --- | --- | --- | --- | --- | --- | --- | --- | --- |
| **Parameters** | **GW1** | **GW2** | **GW3** | **GW4** | **GW** | **GW6** | **GW7** | **GW8** | **GW9** |
| pH | 7.9 | 8.1 | 8.7 | 8.3 | 9.0 | 8.6 | 8.1 | 8.0 | 7.9 |
| EC | 1174 | 1389 | 4125 | 1505 | 1539 | 3687 | 1444 | 1248 | 1318 |
| TDS | 751.4 | 889 | 2640 | 963.4 | 985.2 | 2359.7 | 924.4 | 799.1 | 843.5 |
| Chloride | 374.0 | 543.0 | 930.0 | 335.0 | 289.5 | 860.0 | 373.1 | 361.4 | 407.8 |
| Sulphate | 102.0 | 142.0 | 774.0 | 181.4 | 164.6 | 854.0 | 86.8 | 103.1 | 115.1 |
| Sodium | 122.0 | 50.0 | 665.0 | 228.2 | 270.5 | 362.0 | 180.1 | 92.0 | 120.0 |
| Calcium | 108.0 | 96.0 | 203.0 | 170.0 | 175.6 | 217.0 | 213.8 | 151.1 | 119.9 |
| Magnesium | 56.4 | 44.4 | 100.3 | 67.7 | 65.4 | 105.4 | 99.2 | 56.0 | 49.0 |
| Hardness | 501.2 | 422.0 | 918.7 | 702.5 | 707.2 | 974.6 | 941.3 | 607.3 | 500.6 |
| Nitrate | 27.8 | 36.8 | 40.8 | 38.2 | 43.0 | 39.6 | 52.6 | 45.8 | 41.6 |

Note: All the parameters are in mg/L expect pH and EC in µS/cm.

**Table A29**

Parametric analysis of ground water – May 2016

| **May 2016** | | | | | | | | | |
| --- | --- | --- | --- | --- | --- | --- | --- | --- | --- |
| **Parameters** | **GW1** | **GW2** | **GW3** | **GW4** | **GW** | **GW6** | **GW7** | **GW8** | **GW9** |
| pH | 8.1 | 8.3 | 8.8 | 8.2 | 8.3 | 8.6 | 8.2 | 7.6 | 7.40 |
| EC | 1344 | 1564 | 4360 | 1952 | 1895 | 3994 | 1557 | 1358 | 1422 |
| TDS | 860.2 | 1001 | 2790.4 | 1249.3 | 1212.8 | 2556.2 | 996.5 | 869.4 | 910.00 |
| Chloride | 384.0 | 550.0 | 940.0 | 355.0 | 312.6 | 909.0 | 402.2 | 393.2 | 439.95 |
| Sulphate | 112.0 | 152.0 | 795.0 | 280.0 | 177.8 | 862.0 | 93.6 | 112.2 | 124.18 |
| Sodium | 126.0 | 53.0 | 673.0 | 275.0 | 292.1 | 370.0 | 194.2 | 100.1 | 129.45 |
| Calcium | 112.0 | 99.0 | 201.0 | 159.0 | 189.6 | 225.0 | 230.4 | 164.4 | 129.38 |
| Magnesium | 57.0 | 46.2 | 102.9 | 66.0 | 70.7 | 109.7 | 107.0 | 60.9 | 52.82 |
| Hardness | 513.7 | 436.9 | 924.2 | 668.1 | 763.8 | 1,012.1 | 1,014.8 | 660.8 | 540.01 |
| Nitrate | 28.2 | 37.2 | 41.2 | 38.5 | 46.4 | 40.0 | 56.7 | 49.8 | 44.83 |

Note: All the parameters are in mg/L expect pH and EC in µS/cm.

**Table A30**

Parametric analysis of ground water – June 2016

| **Jun 2016** | | | | | | | | | |
| --- | --- | --- | --- | --- | --- | --- | --- | --- | --- |
| **Parameters** | **GW1** | **GW2** | **GW3** | **GW4** | **GW** | **GW6** | **GW7** | **GW8** | **GW9** |
| pH | 7.9 | 8.1 | 8.6 | 8.4 | 8.2 | 8.4 | 8.3 | 7.7 | 7.5 |
| EC | 1348.0 | 1523.2 | 4250.3 | 2127.7 | 2018.2 | 3898.6 | 1658.2 | 1446.7 | 1514.3 |
| TDS | 862.7 | 974.9 | 2720.2 | 1361.7 | 1291.6 | 2495.1 | 1061.3 | 925.9 | 969.1 |
| Chloride | 375.0 | 540.0 | 920.0 | 387.0 | 332.9 | 890.0 | 428.3 | 418.8 | 468.6 |
| Sulphate | 100.0 | 147.0 | 777.0 | 305.2 | 189.3 | 844.0 | 99.6 | 119.5 | 132.3 |
| Sodium | 125.0 | 51.0 | 655.0 | 299.8 | 311.1 | 358.0 | 206.8 | 106.6 | 137.9 |
| Calcium | 110.0 | 99.0 | 196.0 | 173.3 | 201.9 | 221.0 | 245.4 | 175.1 | 137.8 |
| Magnesium | 59.4 | 45.6 | 100.3 | 71.9 | 75.3 | 108.0 | 113.9 | 64.9 | 56.3 |
| Hardness | 518.5 | 434.5 | 901.2 | 728.2 | 813.5 | 995.1 | 1080.7 | 703.7 | 575.1 |
| Nitrate | 28.4 | 37.4 | 41.4 | 42.0 | 49.4 | 40.2 | 60.4 | 53.1 | 47.7 |

Note: All the parameters are in mg/L expect pH and EC in µS/cm.

**Table A31**

Parametric analysis of ground water – July 2016

| **Jul 2016** | | | | | | | | | |
| --- | --- | --- | --- | --- | --- | --- | --- | --- | --- |
| **Parameters** | **GW1** | **GW2** | **GW3** | **GW4** | **GW** | **GW6** | **GW7** | **GW8** | **GW9** |
| pH | 7.3 | 7.2 | 8.0 | 8.3 | 7.7 | 8.2 | 8.5 | 7.3 | 7.2 |
| EC | 1185 | 1398 | 4065 | 1915 | 1728 | 3705 | 1623 | 1377 | 1454 |
| TDS | 758.4 | 894.7 | 2601.6 | 1225.6 | 1105.9 | 2371.2 | 1039.0 | 881.3 | 930.6 |
| Chloride | 370.0 | 530.0 | 915.0 | 383.1 | 327.9 | 877.0 | 419.3 | 410.4 | 459.2 |
| Sulphate | 94.0 | 142.0 | 769.0 | 302.1 | 186.5 | 840.0 | 97.6 | 117.1 | 129.6 |
| Sodium | 126.0 | 49.0 | 649.0 | 296.8 | 306.4 | 354.0 | 202.5 | 104.4 | 135.1 |
| Calcium | 111.0 | 99.0 | 196.0 | 171.6 | 198.9 | 228.0 | 240.3 | 171.6 | 135.0 |
| Magnesium | 56.4 | 44.4 | 100.3 | 71.2 | 74.1 | 106.3 | 111.6 | 63.6 | 55.1 |
| Hardness | 508.7 | 429.5 | 901.2 | 720.9 | 801.3 | 1005.6 | 1058.0 | 689.7 | 563.6 |
| Nitrate | 28.5 | 37.3 | 41.2 | 41.6 | 48.7 | 40.0 | 59.1 | 52.0 | 46.8 |

Note: All the parameters are in mg/L expect pH and EC in µS/cm.

**Table A32**

Parametric analysis of ground water – August 2016

| **Aug 2016** | | | | | | | | | |
| --- | --- | --- | --- | --- | --- | --- | --- | --- | --- |
| **Parameters** | **GW1** | **GW2** | **GW3** | **GW4** | **GW** | **GW6** | **GW7** | **GW8** | **GW9** |
| pH | 7.20 | 7.10 | 7.90 | 8.30 | 7.70 | 8.10 | 7.80 | 7.30 | 7.20 |
| EC | 1165 | 1345 | 3984 | 1887 | 1694 | 3648 | 1589 | 1349 | 1424 |
| TDS | 745 | 861 | 2550 | 1208 | 1084 | 2335 | 1017 | 864 | 912 |
| Chloride | 365 | 520 | 905 | 379 | 323 | 870 | 410 | 402 | 450 |
| Sulphate | 91 | 140 | 755 | 299 | 183 | 821 | 96 | 115 | 127 |
| Sodium | 122 | 48 | 638 | 294 | 302 | 345 | 198 | 102 | 132 |
| Calcium | 109 | 97 | 192 | 169 | 195 | 222 | 235 | 168 | 132 |
| Magnesium | 55.2 | 46.8 | 98.6 | 70.5 | 73.0 | 103.7 | 109.2 | 62.3 | 54.0 |
| Hardness | 498.8 | 434.3 | 884.2 | 713.7 | 789.2 | 980.1 | 1035.8 | 675.8 | 552.3 |
| Nitrate | 28.20 | 37.00 | 40.90 | 41.17 | 47.98 | 39.70 | 57.89 | 50.98 | 45.86 |

Note: All the parameters are in mg/L expect pH and EC in µS/cm.

**Table A33**

Parametric analysis of ground water – September 2016

| **Sep 2016** | | | | | | | | | |
| --- | --- | --- | --- | --- | --- | --- | --- | --- | --- |
| **Parameters** | **GW1** | **GW2** | **GW3** | **GW4** | **GW** | **GW6** | **GW7** | **GW8** | **GW9** |
| pH | 6.8 | 6.7 | 7.5 | 8.3 | 7.2 | 7.6 | 7.4 | 6.9 | 6.8 |
| EC | 1075 | 1250 | 3787 | 1887 | 2602 | 3429 | 1604 | 1355 | 1428 |
| TDS | 688 | 800 | 2423.7 | 1207.7 | 1665.3 | 2194.6 | 1026.6 | 867.2 | 913.9 |
| Chloride | 345.0 | 490.0 | 856.0 | 379.2 | 678.0 | 827.0 | 410.5 | 402.2 | 450.0 |
| Sulphate | 84.0 | 133.0 | 718.0 | 299.1 | 427.0 | 778.0 | 95.5 | 114.8 | 127.0 |
| Sodium | 113.0 | 40.0 | 606.0 | 293.8 | 376.5 | 323.0 | 198.2 | 102.3 | 132.4 |
| Calcium | 102.5 | 92.0 | 183.0 | 169.9 | 138.0 | 212.0 | 235.2 | 168.1 | 132.3 |
| Magnesium | 46.8 | 45.0 | 95.2 | 70.5 | 57.0 | 98.6 | 109.2 | 62.3 | 54.0 |
| Hardness | 448.1 | 414.5 | 847.8 | 713.7 | 578.7 | 934.3 | 1,035.8 | 675.9 | 552.3 |
| Nitrate | 18.0 | 23.0 | 34.0 | 41.2 | 18.0 | 25.0 | 57.9 | 51.0 | 45.9 |

Note: All the parameters are in mg/L expect pH and EC in µS/cm.

**Table A34**

Parametric analysis of ground water – October 2016

| **Oct 2016** | | | | | | | | | |
| --- | --- | --- | --- | --- | --- | --- | --- | --- | --- |
| **Parameters** | **GW1** | **GW2** | **GW3** | **GW4** | **GW** | **GW6** | **GW7** | **GW8** | **GW9** |
| pH | 6.4 | 6.3 | 7.1 | 6.4 | 6.8 | 7.2 | 7.0 | 6.5 | 6.3 |
| EC | 1034 | 1196 | 3587 | 2325 | 2475 | 3250 | 1700 | 1437 | 1535 |
| TDS | 661.8 | 765.4 | 2295.7 | 1488 | 1584 | 2080 | 1088 | 919.7 | 982.4 |
| Chloride | 330.0 | 470.0 | 811.0 | 570.0 | 643.0 | 786.0 | 443.3 | 432.4 | 488.2 |
| Sulphate | 79.0 | 126.0 | 678.0 | 405.0 | 404.0 | 744.0 | 103.1 | 123.4 | 137.8 |
| Sodium | 107.0 | 37.0 | 581.0 | 335.0 | 359.0 | 303.0 | 214.1 | 110.0 | 143.7 |
| Calcium | 99.0 | 87.0 | 177.0 | 132.0 | 132.0 | 200.0 | 254.0 | 180.7 | 143.6 |
| Magnesium | 50.4 | 43.2 | 91.0 | 57.6 | 54.6 | 95.2 | 117.9 | 67.0 | 58.6 |
| Hardness | 454.1 | 394.6 | 815.4 | 566.2 | 553.9 | 890.3 | 1,118.7 | 726.5 | 599.3 |
| Nitrate | 21.0 | 24.0 | 27.0 | 19.0 | 21.0 | 26.0 | 48.0 | 49.0 | 46.0 |

Note: All the parameters are in mg/L expect pH and EC in µS/cm.

**Table A35**

Parametric analysis of ground water – November 2016

| **Nov 2016** | | | | | | | | | |
| --- | --- | --- | --- | --- | --- | --- | --- | --- | --- |
| **Parameters** | **GW1** | **GW2** | **GW3** | **GW4** | **GW** | **GW6** | **GW7** | **GW8** | **GW9** |
| pH | 6.3 | 6.2 | 7.0 | 6.3 | 6.7 | 7.1 | 6.9 | 6.4 | 6.2 |
| EC | 1022 | 1187 | 3525 | 2295 | 2435 | 3215 | 1778 | 1524 | 1658 |
| TDS | 654.1 | 759.7 | 2256.0 | 1468.8 | 1558.4 | 2057.6 | 1137.9 | 975.4 | 1061.1 |
| Chloride | 322.0 | 462.0 | 800.0 | 562.0 | 635.0 | 770.0 | 469.9 | 462.6 | 446.0 |
| Sulphate | 77.0 | 123.0 | 667.0 | 399.0 | 398.0 | 733.0 | 109.3 | 132.0 | 329.0 |
| Sodium | 105.0 | 35.5 | 570.0 | 329.0 | 353.0 | 290.0 | 226.9 | 117.7 | 139.0 |
| Calcium | 98.0 | 86.0 | 172.0 | 130.0 | 131.0 | 197.0 | 269.3 | 193.4 | 95.0 |
| Magnesium | 49.8 | 42.6 | 89.3 | 56.4 | 54.0 | 94.4 | 125.0 | 71.7 | 39.0 |
| Hardness | 449.2 | 389.7 | 795.9 | 556.2 | 548.9 | 879.3 | 1185.8 | 777.4 | 397.4 |
| Nitrate | 24.0 | 26.0 | 31.0 | 24.0 | 21.0 | 34.0 | 35.0 | 36.0 | 27.0 |

Note: All the parameters are in mg/L expect pH and EC in µS/cm.

**Table A36**

Parametric analysis of ground water – December 2016

| **Dec 2016** | | | | | | | | | |
| --- | --- | --- | --- | --- | --- | --- | --- | --- | --- |
| **Parameters** | **GW1** | **GW2** | **GW3** | **GW4** | **GW** | **GW6** | **GW7** | **GW8** | **GW9** |
| pH | 6.4 | 6.4 | 7.2 | 6.5 | 6.9 | 7.3 | 7.1 | 6.6 | 6.4 |
| EC | 1050.0 | 1245.0 | 3678.0 | 2334.0 | 2502.0 | 3295.0 | 1905.0 | 1625.0 | 1704.0 |
| TDS | 672.0 | 796.8 | 2353.9 | 1493.8 | 1601.3 | 2108.8 | 1219.2 | 1040.0 | 1090.6 |
| Chloride | 326.0 | 464.0 | 826.0 | 569.0 | 644.0 | 790.0 | 502.8 | 495.0 | 460.0 |
| Sulphate | 81.0 | 125.0 | 688.0 | 407.0 | 400.0 | 753.0 | 117.0 | 141.3 | 337.0 |
| Sodium | 110.0 | 39.0 | 590.0 | 336.0 | 365.0 | 301.0 | 242.8 | 126.0 | 142.0 |
| Calcium | 99.0 | 89.0 | 177.0 | 131.0 | 133.0 | 203.0 | 288.1 | 206.9 | 97.0 |
| Magnesium | 51.0 | 43.8 | 91.8 | 57.3 | 55.2 | 96.9 | 133.8 | 76.7 | 39.6 |
| Hardness | 456.6 | 402.1 | 818.9 | 562.4 | 558.8 | 904.8 | 1268.8 | 831.8 | 404.9 |
| Nitrate | 31.0 | 34.0 | 39.0 | 31.0 | 34.0 | 37.0 | 37.5 | 38.5 | 35.0 |

Note: All the parameters are in mg/L expect pH and EC in µS/cm.
